# Abortion providers as human rights defenders: policy priorities for South Africa

**DOI:** 10.3389/frph.2025.1727085

**Published:** 2026-01-12

**Authors:** Lucy Khofi, Jessica Rucell, Mbalenhle Matandela

**Affiliations:** 1 School of Public Health, University of Witwatersrand, Johannesburg, South Africa; 2 Department of Anthropology, University of Amsterdam, Amsterdam, Netherlands; 3 Centre for Applied Legal Studies, University of Witwatersrand, Johannesburg, South Africa; 4 Independent Researcher, Johannesburg, South Africa

**Keywords:** abortion, conscientious objection, health systems, healthcare providers, nurses, reproductive justice, South Africa, policy analysis

## Abstract

South Africa's Choice on Termination of Pregnancy Act (CTOP) protects legal abortion access, yet systemic service delivery barriers persist, limiting care and contributing to preventable maternal morbidity. This policy brief draws on a 2023–2024 mixed-methods study, including a literature review, online survey, focus groups, and in-depth interviews with 33 abortion providers across seven provinces. Findings highlight chronic underinvestment, staffing shortages, unclear conscientious objection guidelines, facility-based stigma, and inadequate managerial support, alongside discrimination against women seeking services. These barriers drive some women toward unsafe alternatives. Despite challenges, providers remain committed, viewing their work as central to reproductive justice and constitutional rights. Addressing gaps requires integrating CTOP into core services, expanding values clarification training, supporting providers, enforcing rights-based guidelines, and engaging communities to ensure safe abortion care.

## Background

South Africa's Choice on Termination of Pregnancy Act (CTOP, 1996; amended 2008) is internationally recognized for enshrining abortion as a constitutional right grounded in dignity, equality, and health. The Act permits termination on request up to 12 weeks and, under specific conditions^1^, up to 20 weeks, demonstrating South Africa's commitment to reproductive autonomy and gender equality.

Despite this strong legal protection, nearly 50% of abortions occur outside designated health facilities, disproportionately affecting poor, rural, and immigrant women, as well as adolescents and girls who face heightened stigma and confidentiality barriers (1, 2). Facility-based stigma, resource constraints, insufficient provider training, and inconsistent application of conscientious objection provisions hinder equitable access. These systemic barriers drive many women to unsafe, unregulated services, increasing maternal morbidity and mortality (2, 3).

While patriarchal and conservative forces have long shaped South Africa's social and political landscape, the current wave of globally connected anti-rights mobilization, including the resurgence of evangelical and conservative actors described by Pilane (4), has gained momentum regionally. This intensifies pressure on an already fragile abortion health system, reinforcing stigma, weaponizing identities, and undermining sexual and reproductive health rights (SRHR). Addressing these systemic barriers is essential to solidifying South Africa's role as a global leader in protecting the bodily autonomy, freedom, and dignity of women and birthing persons.

Founded in 2015, the Sexual Reproductive Justice Coalition (SRJC) is a network of 184 organizations and individuals advancing sexual and reproductive justice in South Africa. Collaborating with clinicians, SRJC works to improve abortion access, expand rights, and hold policymakers accountable to constitutional mandates. Recognizing healthcare providers as essential partners, SRJC conducted a mixed-methods study in 2023–2024 across seven provinces to capture the lived realities, challenges, and insights of providers.

Healthcare provider leadership has proven critical in advancing reproductive rights globally, most recently in Latin America. In South Africa, providers reveal persistent structural barriers, such as inadequate oversight, underinvestment in human resources, and discriminatory practices, that limit safe abortion care. Understanding these challenges through providers' perspectives is vital for strengthening this healthcare service so it better aligns practice with the rights guaranteed by the CTOP Act and the Constitution.

This policy brief synthesizes the legal and social context, provider experiences, and systemic challenges while proposing evidence-based, actionable solutions to bridge the gap between policy and practice. By centering provider insights and strengthening partnerships with healthcare providers to strengthen its healthcare systems, the South African state can confidently reinforce its global leadership in reproductive justice, advancing health equity, and safeguarding the rights and dignity of women and birthing persons in the face of mounting anti-rights threats.

## Problem statement

Despite South Africa's progressive, constitutionally protected legal framework for abortion, structural failures within the health system continue to undermine safe, equitable, and timely access to care. Inadequate investment, staffing shortages, and facility-level stigma drive many, particularly poor, rural, and immigrant women, including undocumented and cross-border migrants, toward unregulated and unsafe operators, increasing preventable maternal morbidity. Weak accountability mechanisms and inconsistent implementation of the CTOP Act deepen these gaps, leaving abortion services vulnerable to anti-rights mobilisation aimed at eroding bodily autonomy and gender equality. Closing these service delivery gaps is essential for realising constitutional commitments and advancing reproductive justice in South Africa.

Complications arising from unsafe and clandestine abortion practices include septic abortion, post-abortal sepsis, severe haemorrhage, uterine perforation, incomplete abortion, pelvic infections, and long-term fertility complications (5). These preventable outcomes place a sustained burden on South Africa's public health system and disproportionately affect women with limited financial means or those facing geographic, social, and informational barriers to accessing timely, safe care.

Women in South Africa seek abortion for diverse and intersecting reasons, including socioeconomic hardship, unsupportive or unstable relationships, intimate partner violence, health risks, the desire to complete education, fear of stigma, and the need to exercise reproductive autonomy (2, 6). These motivations reflect broader structural inequalities that shape reproductive decision-making and underscore the necessity of accessible, rights-affirming abortion care as part of comprehensive sexual and reproductive health services.

Preventing unintended pregnancy remains a critical component of reducing the need for abortion and strengthening reproductive justice. This requires integrated access to a full range of contraceptive methods, youth-friendly services, and comprehensive sexuality education (CSE), alongside structural interventions that address gendered inequalities, poverty, and gaps in information. Strengthening prevention efforts ultimately reduces the burden on an already strained health system and supports women's reproductive autonomy.

In South Africa, public hospitals continue to report high levels of abortion-related morbidity, particularly from incomplete and delayed presentations (7), underscoring the ongoing public health impact of inaccessible or obstructed services. In this policy brief, we align our terminology with the World Health Organization's tripartite classification of abortion as “safe”, “less safe”, and “least safe”. This spectrum reflects the degree of medical oversight involved: clinically supervised CTOP services within the formal health system fall under “safe”, with safety profiles exceeding 90%, while “less safe” and “least safe” abortions typically occur *outside* legal facilities, often through unregulated or illegal operators, self-management without appropriate guidance, or providers lacking the required training or equipment. This framework offers a consistent basis for interpreting the health consequences of restricted or uneven access to legal CTOP services. Regional evidence indicates that 7%–13% of maternal deaths in Sub-Saharan Africa are linked to complications arising primarily from these less safe and least safe procedures (8, 9).

## Methodology

This policy brief draws on an assessment conducted by the Sexual and Reproductive Justice Coalition (SRJC) between June and September 2023 to document abortion healthcare providers' perspectives on barriers and solutions to expanding safe abortion access in South Africa. The project was undertaken as an internal needs assessment for programmatic planning; therefore, formal ethics approval was not required (10). Nevertheless, all ethical principles, including informed consent, confidentiality, participant autonomy, and secure data handling, were strictly observed.

The assessment aimed to:

identify key structural and operational barriers affecting the delivery of abortion services, and
examine providers' experiences, roles, and recommendations for strengthening access.

## Study design

To meet these objectives, the study employed a three-tiered mixed-methods design to ensure representation across provinces, clinical roles, levels of experience, and facility contexts. The sequential structure enabled each tier to inform the next: survey findings shaped the focus group discussion (FGD) guide, and FGD insights guided the purposive selection of in-depth interview (IDI) participants and the refinement of interview priorities.

The design comprised three interconnected components. First, a desktop review of peer-reviewed literature, Department of Health reports, and grey literature provided the contextual foundation for understanding abortion provision in South Africa. Second, empirical data were collected through an online survey completed by SRJC-affiliated abortion providers. Third, qualitative data were gathered through five focus group discussions (FGDs) with survey respondents, followed by six in-depth interviews (IDIs) with a purposively selected subset of participants to ensure variation across provinces, clinical roles, and years of experience. This integrated and iterative approach enabled triangulation and facilitated deeper exploration of emerging themes.

## Recruitment

Participants were recruited through SRJC mailing lists, SRJC WhatsApp provider groups, and coordinated outreach by provincial SRJC provider leads, facilitating participation across diverse provincial and facility contexts. Midwives, nurses, and doctors involved in providing or supporting Choice on Termination of Pregnancy (CTOP) services were invited to participate, ensuring varied professional insights into health system dynamics.

## Participant sample & ethical protocol

A total of 33 providers completed the online survey, which examined barriers to CTOP provision, facility-level stigma, interpretations of conscientious objection, stock-outs of essential medicines, provider burnout, and workplace support structures. Participants for the FGDs and IDIs were selected from survey respondents using criteria designed to ensure variation in province, clinical role, and years of experience.

The final sample comprised 31 women and 2 men, including midwives, nurses, and doctors working across seven of South Africa's nine provinces. All participants received a study information sheet and provided digital consent before participating. Ethical principles, voluntary participation, confidentiality, respect for autonomy, and secure data protection were upheld throughout all stages of data collection.

## Analysis

Quantitative data was analysed descriptively, while qualitative data was examined through narrative and framework approaches to identify barriers and pathways to strengthening safe abortion access.

Qualitative analysis followed a combined narrative and framework approach. First-cycle coding involved descriptive, process, and values-based codes, which were then organised into a framework matrix to compare themes across provinces, clinical roles, public and private sectors, and facility types. Narrative analysis guided the interpretation of providers' stories, centring their lived experiences. To enhance institutional participation and analytic rigour, a subset of transcripts was independently reviewed by two SRJC staff members, with coding differences reconciled through discussion. This process ensured consistency and strengthened the interpretive validity of the findings.

## Findings and discussion

Findings from the study reveal that systemic barriers continue to undermine the implementation of South African's right to safe abortion, despite its progressive legal framework. Overall, providers reported that inadequate resourcing and chronic staffing shortages impede timely, quality abortion care across most provinces. Heavy workloads, insufficient clinical, managerial, and administrative support, and limited access to mentorship and debriefing contribute to delays and exacerbate provider burnout, further constraining service availability (11).

Although the CTOP Act has historically been interpreted to allow limited conscientious objection, applying only to direct participation in the procedure, the emerging position of the National Department of Health (NDoH) clarifies that such refusals are not compatible with the Act's obligations to ensure timely access to care. In practice, providers who object have nevertheless been expected to fulfil related duties, including referral, counselling, and ensuring continuity of care, but the study found that managers and administrators frequently misinterpret this clause, resulting in broad, facility-wide refusals that go far beyond what the law intended. These misinterpretations contribute to avoidable delays, inconsistent service availability, and violations of women's constitutional rights to health and reproductive autonomy (12). The forthcoming NDoH CTOP guidelines, currently under revision, are expected to formalise this clarification and reinforce provider and managerial accountability for ensuring uninterrupted access to legal services.

Our findings affirm existing literature on stigma and discrimination. Both note discrimination in health facilities further deters access to abortion care. Providers frequently face derogatory remarks, judgment, and refusal of duties from colleagues, creating hostile work environments that discourage participation in abortion provision and perpetuate negative attitudes toward patients seeking services (13). This contributes to cultures of isolation, silence, and avoidance, further limiting the reach of CTOP services.

As one provider explained, “Colleagues tell us we are killing babies, and they refuse to support the service even when the law is clear”. [Professional Nurse (Midwife), 11 years' experience, Gauteng].

Another shared, “Managers act as if CTOP does not exist; they avoid scheduling the service or pretend there is no demand”. [Professional Nurse (Midwife), 7 years' experience, Eastern Cape].

A third provider reflected, “The stigma comes more from inside the facility than from the community”. [Medical Officer (Doctor), 4 years' experience, Western Cape].

These accounts highlight how peer-level stigma and managerial avoidance shape providers' day-to-day working environments. Because abortion provision in South Africa is predominantly carried out by women, especially midwives and Professional Nurses, its devaluation reflects broader patterns of gendered discrimination that persist in the democratic era. Participants described how midwives often shoulder the full clinical and administrative burden of CTOP services, while many colleagues (including other midwives, assistant nurses, and support staff) are permitted to refuse abortion-related tasks by invoking conscientious objection. As one Professional Nurse (Midwife) with 9 years' experience in KwaZulu-Natal explained, “*When it comes to CTOP, suddenly everyone has an objection. They refuse to even prepare the room. Everything falls on us*”. Another Professional Nurse (Midwife) with 12 years' experience in the Free State shared, “*Management knows people are refusing, but they look away. They say, “we can't force them”, so the whole service becomes our responsibility*”. Providers across provinces reported that managers and hospital heads frequently condone or inconsistently enforce these refusals, in ways misaligned with policy, reinforcing a system in which feminised labour is undervalued and insufficiently supported.

This creates uneven workloads, reinforces discrimination and institutional silence around abortion services, and contributes to moral distress among providers who, despite this, remain committed to upholding their ethical duties as clinicians under the Hippocratic Oath. These intra-facility dynamics deepen existing systemic barriers and undermine the full implementation of the CTOP Act.

This poor interpretation of conscientious objection significantly disrupts workflow within facilities by causing delays in care, fragmenting clinical teams, and increasing the workload on non-objecting staff. These disruptions undermine scheduling, weaken emergency preparedness, and contribute to provider burnout. The inconsistent application of conscientious objection also creates uncertainty for clients and staff, further affecting the reliability and efficiency of CTOP services across both public and private-sector facilities, where limited staffing and inconsistent managerial oversight can similarly amplify the impact of refusal.

Persistently poor information on the legality and availability of services, along with the continued existence of unsafe and illegal abortion operators, remains a critical concern. Many girls and women, particularly those who are poor or living in rural areas, are unaware of the availability of safe legal abortion services and are driven to seek care from unregulated operators due to a lack of accessible information and safe options. These unsafe procedures increase the risk of severe health complications, including maternal death. While South Africa lacks comprehensive national data, regional evidence shows the significant contribution of unsafe abortion to maternal morbidity and mortality across Sub-Saharan Africa (14).

Our analysis also highlights disparities between the public and private sectors. While the public sector faces chronic stigma, resourcing constraints, managerial gaps, and limited training opportunities, private providers report greater operational independence but experience regulatory challenges and inconsistent collaboration with public facilities. The cost of private sector services remains a significant barrier, with medical abortions costing approximately R1,700–R1,800 ($94–100 USD) and surgical abortions ranging from R1,500 to R7,410 ($83–413 USD), depending on gestational age and facility. Given that the median monthly earnings for South African women are approximately R4,000 (Stats SA, 2023), even the lowest-cost private-sector abortion can consume nearly half of a woman's monthly income, making private care inaccessible for most and deepening inequities in access. Although medical insurance schemes are legally required to cover CTOP under Prescribed Minimum Benefits (PMB), uneven application and high out-of-pocket expenses persist, limiting equitable access (15).

Working women, adolescents, and migrant women, especially, try to afford private services, due to increased confidentiality, the potential of less anti-foreigner sentiment, long waiting times, stigma, limited staffing, and unpredictable service availability within the public sector. These patterns reflect broader structural inequalities and demonstrate how uneven implementation of the CTOP Act continues to shape differentiated access to safe, timely abortion care.

Some private facilities rely more heavily on medical management between 12 and 20 weeks of gestation to minimize the administrative and documentation requirements associated with surgical procedures. While this approach can reduce facility burden, the multi-day nature of medical management requires additional follow-up and support to ensure safety, as noted in the WHO abortion care guidelines (16).

We found healthcare providers position themselves as champions of reproductive justice, viewing their role as essential to advancing women's rights and autonomy. Many frame abortion provision as a form of resistance to sexism, demonstrating commitment to upholding constitutional rights and addressing health inequities despite discrimination and inadequate institutional support (11).

Survey responses and FGD revealed a clear consensus: the current abortion health system is not meeting the legal and ethical standards set by South Africa's laws and Constitution. Providers articulated the urgent need for systemic reforms, including improved facility resourcing, clear enforcement of conscientious objection guidelines, stigma reduction within health systems, and within the public sector, consistent supply chains for essential medicines. Additionally, the study identified opportunities to expand safe abortion services by scaling up self-managed medical abortions (SMA), which are less costly, more independent, and compliant with the existing regulatory system.

These insights show the critical role providers play in bridging the gap between policy and practice. Their experiences offer a grounded understanding of systemic shortcomings and point to where targeted investments and policy changes can have the greatest impact. Leveraging these insights can guide actionable reforms to advance reproductive justice, reduce preventable maternal morbidity, and ensure that South Africa's progressive legal commitments translate into equitable, safe abortion access for all who need it.

Building on providers' insights, the study identified practical opportunities to strengthen abortion service delivery within South Africa's health system. The table below summarises the key systemic challenges highlighted by providers alongside actionable solutions they propose to advance safe, equitable, and rights-based abortion care (see Table 1 above).

**Table 1:** Policy challenges, solutions and actions.

Challenge	Solutions proposed by providers	Priority actions required
1. Lack of government and departmental support & resourcing	Integrate CTOP into essential SRH and NHI service packages, especially at PHC level.	Normalise CTOP as a core component of basic human rights and reproductive healthcare. Strengthen departmental leadership and budget allocation.
2. Lack of managerial, facility, mentorship, and collegial support	Implement VCAT for all MCWH provincial, district, and facility managers. Train supervisors in gender-responsive planning and budgeting.	Strengthen supervisory training, monitoring, and accountability. Ensure managers fulfil mandated duties. Facilitate structured mentorship for CTOP providers.
3. Limited clinical and support staffing	Increase remuneration and improve staffing norms to match service demand.	Advocate for professionalisation and sustainable funding for CTOP providers through partnerships with unions, NGOs, and professional associations.
4. Societal stigma	Change societal norms through sustained VCAT, public education, and attitude-transformation initiatives.	Implement national public information campaigns; support local community outreach involving DoH, DSD, DoE, TVET institutions, providers, and community leaders.
5. Facility-based discrimination	Budget for bi-annual VCAT and structured debriefing sessions across all provinces and districts.	Strengthen training, monitoring, and accountability mechanisms. Ensure compliance through district health management teams and partners such as IPAS.
6. Illegal back-street operators	Develop a clear national strategy for identifying, policing, and reducing illegal abortion practices.	Conduct targeted investigations, arrests, and legal action through SAPS, DoJ, Public Works, and local authorities. Expand safe, public-sector CTOP access as deterrence.
7. Provider isolation and burnout	Establish a peer-provider debriefing network with dedicated budget support.	Hold quarterly provider reflection and support meetings through Departments of Health, unions, NGOs, and SRHR networks.

## Policy implications

To close the implementation gap and fully realise South Africa's constitutional duties to reproductive rights, systemic strengthening across facility oversight, accountability mechanisms, staffing, and stigma reduction is essential. Providers across the country agree on where targeted interventions can have the greatest impact. These include ensuring the CTOP Act is not only legally protected but reliably implemented as part of routine sexual and reproductive healthcare. These reforms are critical for moving from a framework of rights on paper to meaningful, equitable realisation in practice.

We recommend the following:

Invest in services and staff: Increase remuneration, mentorship, training, and structured debriefing to ensure quality care and address provider burnout.
Align law and policy: Strengthen and clarify national guidance on the role and limits of conscientious objection to ensure consistent and lawful implementation of the CTOP Act. Clear accountability mechanisms are essential for uninterrupted service delivery. Potential approaches include written documentation of refusal, routine CTOP service audits, supervisory reporting requirements, and facility-level monitoring systems to prevent unlawful obstruction of care and ensure timely availability of services. International experiences offer useful examples: countries such as Uruguay and Mozambique have introduced structured oversight systems, including documentation requirements, mandatory referral pathways, and routine monitoring, to minimise service disruption and improve access.
Enhance public sector SRH services: Integrate CTOP into broader SRH services under Primary Health Care (PHC) and National Health Insurance (NHI) model, with standardised monitoring, support for staff transitions and retirements, and structured onboarding of new providers.
End stigma: To shift entrenched negative attitudes, implement bi-annual Values Clarification and Attitude Transformation (VCAT)^2^ training and anti-discrimination training for provincial, district, and facility managers, as well as for community health workers. Participants across all provinces unanimously emphasised the positive impact of VCAT on improving team dynamics and reducing stigma within their facilities.
Foster collaboration and peer support: Establish digital and in-person peer support networks among CTOP providers, and promote public–private collaboration on monitoring, service delivery, and sharing of best practices.
Ensure medication and information availability: Strengthen procurement and consistent distribution of Essential Medicines List (EML) commodities, and provide clear, accessible information on CTOP services and the dangers associated with illegal and unregulated abortion providers.
Strengthen prevention: Expand comprehensive sexuality education (CSE), youth-friendly contraceptive services, and community-based safe-sex initiatives to reduce unintended pregnancy and support reproductive autonomy.

## Footnotes

^1^These conditions include: (1) risk to the pregnant person's physical or mental health, (2) significant fetal abnormality, (3) pregnancy resulting from rape or incest, or (4) the continued pregnancy significantly affecting the pregnant person's social or economic circumstances (CTOP Act, 1996; Amendment Act, 2008).

^2^VCAT is an evidence-based training approach that supports providers to reflect on personal beliefs, reduce stigma, and strengthen rights-based abortion care. Evidence from South African and global VCAT programmes consistently shows meaningful and sustained improvements in provider attitudes and willingness to support CTOP implementation.
